# Advances in Polymer Nanocomposites for Drilling Fluids: A Review

**DOI:** 10.3390/ma18204809

**Published:** 2025-10-21

**Authors:** Shahbaz Wakeel, Ammara Aslam, Jianhua Zhang

**Affiliations:** 1 Key Laboratory of Systems Bioengineering of the Ministry of Education, Department of Polymer Science and Engineering, School of Chemical Engineering and Technology, Tianjin University, Tianjin 300350, China; shahbazwakeel410@tju.edu.cn (S.W.); ammaraaslam18@gmail.com (A.A.); 2 Tianjin Key Laboratory of Membrane Science and Desalination Technology, Tianjin University, Tianjin 300350, China

**Keywords:** drilling fluids, polymeric filtrate reducer, shale swelling, polymer nanocomposites, temperature resistance, salt tolerance

## Abstract

Hydrocarbon exploration and extraction increasingly rely on drilling fluids that guarantee operating safety and efficiency, particularly in ultra-deep, high-temperature, and unconventional reservoirs. Traditional drilling fluids, especially for water-based muds (WBMs), have several problems, including excessive fluid loss, severe swelling in shale and instability in high-pressure/high-temperature (HPHT) conditions. Polymer nanocomposites (PNCs) are new types of drilling fluid additives that combine the vast surface area and reactivity of nanoparticles (NPs) with the structural flexibility and stability of polymers. This combination enhances rheology, reduces filtrate loss, and, most importantly, creates hydrophobic and pore-blocking barriers that prevent shale from swelling. This review highlights important improvements in drilling fluids with PNCs regarding exceptional rheological properties, low fluid loss, and improved suppression of the shale swelling. The particular focus was placed on the specific mechanisms and role that PNCs play in enhancing shale stability, as well as their responsibilities in improving rheology, heat resistance, and salt tolerance. Current advancements, persistent hurdles, and prospective prospects are rigorously evaluated to emphasize the scientific and industrial trajectories for the development of next-generation, high-performance drilling fluids. Moreover, the current challenges and future opportunities of PNCs in drilling fluids are discussed to motivate future contributions and explore new possibilities.

## 1. Introduction

**Figure 1:**
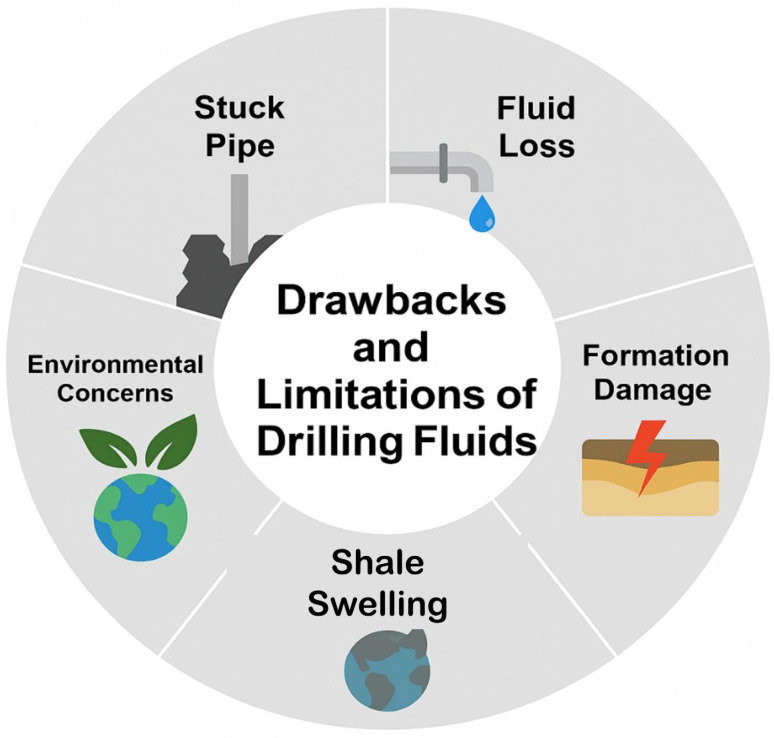
Drawbacks and limitations of drilling fluids.

Drilling fluids, often referred to as drilling muds, play a crucial role in oil and gas exploration by controlling pressure in the hole, transporting cuttings, lubricating the drill bit, and maintaining the wellbore stability. They are primarily divided into oil-based muds (OBMs) and water-based muds (WBMs). OBMs are relatively stable in terms of heat and chemicals, but they are detrimental to the environment and the economy because they are poisonous and expensive to clean up [1]. WBMs have become preferred because of their lower environmental impact and lower disposal costs. Still, they have operational issues, such as losing too much fluid, expanding shale, and being unstable in high-pressure/high-temperature (HPHT) conditions. Figure 1 illustrates the important issues of typical drilling fluids, including fluid loss, instability at high temperatures, and shale swelling. To resolve the above problems and achieve better performance of drilling fluids during drilling operations in complex geological conditions of deep formations, some additives have been developed and widely employed to change the physicochemical properties and improve the applicable properties of drilling fluids, especially for WBMs. However, the complex geological conditions in deep formations with high temperature, high pressure, and high salinity result in high requirements for the performance of drilling fluids. Therefore, it is imperative to explore a new methodology and techniques to accelerate the search for more effective WBMs and related additives to address these issues of WBMs and ensure the efficient drilling and exploration of oil and gas resources mainly distributed in deep layers [2].

Various additives are demonstrated to decide the physicochemical properties and functional characteristics of WBMs. The additives have become indispensable, especially for drilling activities in deep formations, unconventional reservoirs, and harsh circumstances. The functional additives serve critical functions such as minimizing fluid loss, maintaining wellbore stability, ensuring drilling safety and mitigating environmental impacts. Polymeric WBMs improve drilling fluid rheology, fluid loss, and wellbore stability. Shale swelling, low ROP, and formation damage plague traditional WBMs. They benefit from natural or synthetic additions. Despite their benefits, polymeric additives must be examined for cost, environmental impact, and drilling site characteristics. Table 1 not only compares the pros and cons of natural and manufactured polymers, but also describes the primary functions, and operational limitations of polymeric additives utilized in drilling fluid formulations. Biopolymers such as XG, GG, and starch exhibit favorable rheological modification capabilities, ecological compatibility, and effective fluid loss mitigation; however, they are often constrained by thermal degradation, microbial susceptibility, and limited solubility under high-pressure, high-temperature (HPHT) conditions. Most natural polymers fail at 150 °C or higher hot drilling temperatures due to thermal degradation. Many modification strategies have been researched to increase natural polymer performance in high-temperature situations [3,4]. Ionic crosslinking, esterification, etherification, salinization, hydroxymethylation, and free radical polymerization were studied. Modified natural and synthetic polymers, including HPAM, KPAM hydrogel, PHPA, PAC, HEC, and CMC, demonstrate enhanced thermal stability, shale inhibition, and improved rheological control. Still, their performance can be compromised by ion sensitivity, elevated viscosity, and environmental concerns related to monomer toxicity. Advanced polysaccharides such as chitosan, scleroglucan, welan gum, and schizophyllan offer promising viscoelastic behavior, salt tolerance, and biodegradability. Yet, they remain prone to oxidative degradation and reduced structural integrity under biologically active or thermally extreme environments. The optimal selection of these additives must therefore consider the thermodynamic and geochemical constraints of the target formation to ensure functional efficiency, environmental safety, and economic viability [5]. Synthetic additives like polyacrylate (PAA) and PAM were introduced to improve the performance of fluids. Adding these chemicals enhanced the filtering capabilities and viscosity of water-based drilling fluids (WBDFs), rendering them more adept at enduring high-temperature and deep-well drilling conditions [6]. However, these improvements were not enough to solve WBDFs problems with performance instability, especially during changing drilling conditions with different temperatures, pressures, and salt levels [7].

**Figure 2:**
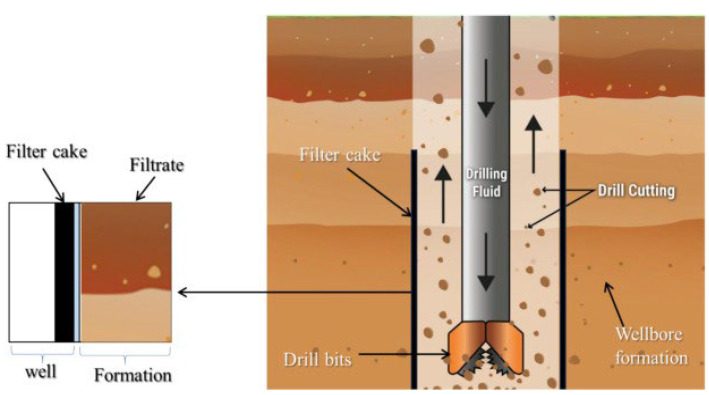
Schematic representation of filter-cake microstructure in conventional and nanoparticle-enhanced water-based drilling fluids (WBDFs) [26].

Parallel to the aforementioned developments in polymer additives, nanomaterials as one of the most promising materials have attracted enormous interest and achieved substantial progress for a diverse array of applications in various fields in recent decades, due to their nano-sized characteristics, tunable morphology, versatile modification possibilities, unique physicochemical properties and well-defined multifunctionalities. Various nanoparticles (NPs) have also exhibited great potential in drilling fluids [8,9]. For example, inorganic nanoparticles such as SiO_2_, mesoporous silica nanoparticles (MSNs), and clay, help stabilize shale by making it less permeable and stronger as a filter cake. Their tiny size allows them to effectively plug micro- and nanopores, creating a denser and less permeable barrier that stops filtrate invasion and improves wellbore sealing. This action makes shale minerals less hydrated, thereby increasing the wellbore’s stability. Alongside shale stabilization, integrating NPs with lost circulation materials has shown efficacy in reducing filtrate loss in drilling fluids [10,11]. Including NPs in inversion emulsion muds, in conjunction with low-cost additives, greatly reduced filtrate loss and yielded a thinner filter cake than muds containing just NPs or low-cost additives alone. NPs reduce filter cake permeability by occupying interstices between nano- and micron-sized particles. Integrating NPs into the drilling fluid produces a more consistent and denser filter cake, reducing fluid loss and enhancing the overall efficacy of the drilling process. Figure 2 demonstrates that the incorporation of NPs decreases filtrate loss volume and improves the sealing of the filter cake in comparison to conventional drilling fluids devoid of NPs. The image illustrates the disparities in fluid loss behavior between muds containing NPs and those without, showcasing the efficacy of NPs in reducing filtrate loss during drilling operations [12,13]. Despite these attractive features, NPs are associated with difficulties related to stability and aggregation. Due to their increased energy density and small sizes, NPs are prone to aggregation in the mud system. Such an inclination to aggregate is due to strong interactions, such as metallic or covalent contacts, and weaker van der Waals interactions that cause groupings of particles [14].

Currently, the development of drilling fluid technology has increasingly centered on polymer nanocomposites (PNCs), which amalgamate the elevated surface reactivity of nanoparticles (NPs) with the stability and structural adaptability of polymers. This synergy enables PNCs to outperform single polymers or nanoparticles by reducing the amount of filtrate lost, enhancing the material’s flow, and resisting damage from heat and salt [15]. The polymer matrix is important because it prevents nanoparticles from clumping together, ensuring they are evenly distributed and remain stable over time. Furthermore, the NPs improve the performance of the polymer because of the molecular chain entanglement upon adsorption onto the polymer matrix surface [16]. There are multiple mechanisms through which interaction between NPs and polymers may occur, including electrostatic attraction, hydrophobic interactions, steric repulsion, hydrogen bonding, and electrostatic repulsion [17,18]. These interactions result in new nanocomposite materials that are extraordinary and often exhibit important improvements compared to their building blocks. These enhanced characteristics include advanced performance, resistance to hostile temperatures, and the ability to withstand increased salinity levels [19]. Across the studies summarized in Table 1 and Table 2, PNC additives generally increase YP relative to base WBM and lower HPHT filtrate loss under saline/thermal stress; the magnitude depends on NP type/size and surface treatment, with silica- and graphene-based systems most consistent under high salinity [20]. For many of the issues that traditional drilling fluids cause, PNCs offer a consistent fix. PNCs can increase wellbore stability and dramatically slow fluid loss [21]. For example, shale swelling remains one of the most important challenges in drilling operations. It often leads to wellbore instability, pipe sticking, and structural collapse. Under HPHT and saline conditions, conventional inhibitors such as potassium chloride or simple polymers provide only partial protection. PNCs mitigate these effects through two complementary mechanisms: (i) forming nanopore-blocking barriers that minimize filtrate invasion, and (ii) generating hydrophobic layers that reduce water uptake and the resulting volumetric expansion of shale minerals. PNCs can also provide improved thermal stability, guaranteeing that the drilling fluid is proper even under demanding circumstances [22,23]. Better lubricating qualities of PNCs also help lower the coefficient of friction (CoF) between the drill bit and the wellbore, lowering equipment wear and increasing efficiency. Reduced fluid loss and enhanced sealing capacity produced by the synergy between the NP and the polymer matrix also help avoid fluid intrusion into adjacent formations and strengthen wellbore integrity [24,25]. In sum, polymer nanocomposites (PNCs) offer a reliable and technically sound solution to many of the challenges associated with traditional drilling fluids, including excessive filtrate loss, low temperature resistance, and shale swelling. Through the synergistic interaction between nanoparticles and polymer matrices, PNCs enhance rheological stability, reduce filtrate invasion, and strengthen wellbore sealing.

This review aims to summarize and rigorously assess the most recent advancements in the formulation, processes, and practical applications of PNCs-based drilling fluids. It specifically looks at (i) modification strategies and how they affect rheological behavior, fluid-loss control, and thermal stability; (ii) the role of inorganic and metallic nanoparticles in improving wellbore integrity and filter cake structure; and (iii) the synergistic mechanisms by which PNCs improve shale inhibition and drilling efficiency. We believe that this review article will offer some ideas on the design and use of PNCs for researchers working in this area.

## 2. Overview of PNCs

Drilling fluids serve several essential functions, including transporting cuttings, controlling pressure, stabilizing the wellbore, and preventing fluid from escaping. All of these things are more challenging to accomplish under HPHT and saline conditions. PNCs have become advanced, multifunctional additives that can help address these problems. Their unique polymer–nanoparticle synergy enables more effortless movement of cuttings, maintains viscosity and gel strength, makes the material resistant to heat, and creates nano barriers that prevent filtrate invasion and shale swelling. This section discusses the formation, structure, and operation of PNCs in drilling fluids.

### 2.1. Fundamentals and Preparation of PNCs

PNCs are hybrid materials made of nanoparticles (NPs < 100 nm) that are spread out in a polymer matrix. This makes them better at filtering, rheology, and thermal properties. The polymer matrix ensures that the nanoparticles are evenly distributed and stable. The nanoparticles add more surface activity, barrier strength, and chemical resistance.

NPs are incorporated into a polymer matrix using a variety of processes during the manufacturing of PNCs to guarantee a uniform distribution and optimal performance of the nanocomposite material. The most often used techniques for PNC preparation with their impact on shale stability and inhibition are represented in Table 2.

The Solution Mixing technique dissolves the polymer in a solvent and distributes the NPs among the polymer solution. By following dispersion, the solvent evaporates, forming a solid nanocomposite layer or substance. Although this method guarantees good dispersion of the NPs inside the polymer matrix, it may make it challenging to maintain their stability in the solution.

The melt mixing method combines the polymer with NPs in the melt state, in which the polymer is heated above its melting point. Mixing the NPs with the molten polymer guarantees their uniform distribution over the matrix. Thermoplastic polymers are processed mostly using this technique since it is reasonably affordable and appropriate for mass manufacturing. Still, it can be challenging to regulate the dispersion of NPs in the molten form.

In situ polymerization involves the direct polymerization of monomers when NPs are present. Clusters of polymer chains form around dispersed nanoparticles, including SiO_2_, TiO_2_, CNTs, GO, ZnO, and other metal nanoparticles (such as gold or silver). Among the several advantages of this approach are the high dispersion and robust interactions between the nanoparticles and the polymer. In many cases, nanoparticles initiate or accelerate the polymerization reaction, which in turn strengthens the contact. Nanocomposites made of thermoset-based polymers are commonly created using this method. As a result, the composite’s mechanical, chemical, thermal, and adhesive properties are all enhanced. Every preparation technique has benefits and drawbacks; the type of polymer, NPs, and desired qualities of the resultant nanocomposite material will determine the method of choice. These preparation techniques aim to ensure homogeneous dispersion of the NPs inside the polymer matrix, thereby ensuring that both components’ synergistic benefits are completely realized and producing a material displaying exceptional performance under demanding conditions [27]. PNCs are a type of high-tech material that can contain both organic and inorganic nanoparticles. The primary factor determining the type of PNC is the type of nanoparticles employed.

#### 2.1.1. Strategic Integration of Polymers and Nanoparticles for Advanced Drilling Fluids

The type of polymers and NPs best suited for drilling depends on the environment in which the drilling will take place. Polyanionic cellulose (PAC) and carboxymethyl cellulose (CMC) are environmentally friendly and help manage viscosity. Polyacrylamide (PAM) and AMPS copolymers enhance the material’s resistance to salt and temperature fluctuations. Inorganic nanoparticles (SiO_2_, clays, MSNs) make the filter cake denser and seal the pores more effectively. Metal and metal oxide nanoparticles (TiO_2_, ZnO, Fe_3_O_4_) enhance the material’s resistance to heat and improve its ability to control fluid loss by crosslinking and strengthening the structure. This combination of organic polymers and inorganic/metallic nanofillers enables the creation of next-generation drilling fluids that perform well under varying field conditions and maintain their rheological and filtration properties.

#### 2.1.2. PNCs Made of Polymers and Inorganic NPS

Inorganic NP_S_ are widely used in PNCs to enhance the tensile strength, chemical resistance, and thermal stability of the composites. These NPS, including SiO_2_, quantum dots (QDs), and clays, are particularly beneficial under demanding conditions, improving the viscosity, wellbore stability, and filtration control of composite materials. Inorganic NPS play a crucial role in making PNCs suitable for a range of industrial applications, from structural composites to wellbore drilling fluids.

Silica NPS are among the most commonly used inorganic NPS due to their large surface area, good thermal stability, and biocompatibility. These properties allow SiO_2_ to enhance the mechanical, viscosity, and chemical resistance of composites. SiO_2_ NPS are widely used in structural composites, medicinal applications, WBMs, and lubricants, contributing to their overall performance. Additionally, mesoporous silica nanoparticles (MSNs) are ideal for drug delivery and catalysis applications due to their high surface area and adjustable pore diameters. These features make MSNs useful in adsorption systems, catalysis, and controlled drug delivery [8,27].

#### 2.1.3. PNCs Made of Polymers and Metal NPS or Metal Oxide NPS

In PNCs, metal and metal oxide NPS are used to enhance mechanical strength, thermal stability, and electrical conductivity. These NPS also possess biocidal and catalytic properties, making them ideal for applications in biomedical devices, electronics, and catalysis. Standard metal and metal oxide NPS in PNCs include Cu, Ni, Co, Pt, Ag, Au, Fe_3_O_4_, Fe_2_O_3_, TiO_2_, and ZnO. Metallic NPS, such as copper, nickel, and gold, improve the electrical conductivity, catalytic activity, and biocompatibility of PNCs. These properties make them useful in various applications, including electronic devices, drug delivery systems, and biosensors. The high conductivity and catalytic properties of metallic NPS enable advancements in fields such as electronics and healthcare. Platinum and silver are also commonly used for their unique catalytic and biocidal properties, making them essential in biomedical applications and environmental remediation [28,29,30].

#### 2.1.4. Properties and Advantages of PNCs

Drilling fluids can be greatly improved with the use of PNCs, particularly in extreme drilling conditions. NPSs, such as Gr, SiO2, or clay, are added to PNCs to enhance valuable properties, including resistance to heat and salt. For drilling fluids to remain stable and perform well in extreme situations, such as the high temperatures and salty environments encountered during deep drilling, these qualities are crucial. Because it is now more resistant to heat, the drilling fluid will retain its properties even in extremely harsh environments. Because of its enhanced salt resistance, the fluid can be used effectively in areas with high salt concentrations. Additionally, PNCs can thicken drilling fluids, which is crucial for controlling the viscosity of the fluid [31,32,33]. The enhanced thickening ability facilitates the suspension of drill cuttings and other materials, leading to streamlined and expedited drilling operations.

Drilling fluids treated with PNCs are not only more resistant to cold and salt, but their properties can be fine-tuned by adjusting the nanoparticle type and concentration. Due to this adaptability, tailored fluids that perform effectively in various drilling conditions can be developed. Drilling fluids that contain PNCs not only perform better, but they also reduce the likelihood of wellbore instability and improve the whole operation’s efficiency. PNCs can alter the flow of fluids, strengthen suspensions, and increase the stability of fluids in harsh environments, all of which contribute to improved drilling fluid performance in challenging scenarios.

By including NPS like GO, CNTs, and SiO_2_, PNCs can increase the fluid’s viscosity and yield point. Cuttings can be suspended and moved more easily as a result. This alteration is necessary to enable wellbore stability and efficient hole cleaning. Additionally, the presence of NPS helps avoid issues such as shale swelling and wellbore instability, which can be costly and time-consuming to rectify. The construction of a protective barrier by the NPS stabilizes the wellbore and reduces fluid leakage into the formation, thereby further enhancing the well’s integrity [23,34,35].

### 2.2. PNCs in Drilling Fluids

PNCs have demonstrated good performance in drilling fluid effectiveness, and adding NPs to a polymer matrix enhances fluid loss, wellbore stability, and filter cake formation in drilling operations. Instead of basic polymers, PNCs combine the benefits of the polymer matrix with NPs to improve mechanical, thermal, and chemical performance [36,37,38].

In drilling fluids, the primary function of PNCs is to reduce filtrate loss, which is crucial for maintaining wellbore integrity. Traditional drilling fluids often encounter issues such as fluid loss into the formation, which can lead to instability and potential operational problems. By adding NPS to polymer matrices, PNCs fill the gaps between nano- and micron-sized particles, creating a denser and more uniform filter cake. This helps to prevent fluid loss, maintaining the efficiency of the drilling operation. Studies have shown that PNCs, when combined with low-cost additives, greatly reduce fluid loss and form thinner filter cakes compared to fluids with just NPS or additives alone.

An important challenge for PNCs, however, is the stability and aggregation of the NPS within the fluid. Due to their small size and high surface energy, NPS tend to aggregate, which can compromise their effectiveness. Aggregation causes a reduction in the fluid’s viscosity and alters its thermal properties, negatively impacting the overall performance of the drilling fluid. To address this, surface modification techniques have been developed to enhance the dispersion and stability of NPS in the polymer matrix. These modifications include the use of surfactants, polymers, and silane functional groups, which prevent aggregation and ensure the NPS remain uniformly distributed throughout the fluid [39,40]. These enhancements allow PNCs to maintain their performance, even under challenging HPHT conditions.

**Figure 3:**
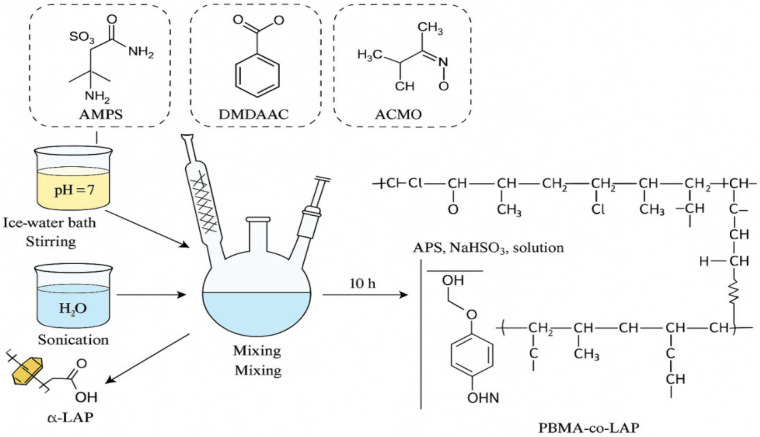
Schematic diagram of process steps for developing PNCs PDADA-LAP for drilling fluid. Reproduced with permission [40]. Copyright 2024, Elsevier.

In the most recent Study, in situ polymerization technologies are being used to create unique PNC additives that can help with drilling situations that are becoming increasingly challenging. Figure 3 illustrates the synthesis of α-laponite (α-LAP) clay-based polymer nanocomposites through in situ polymerization of monomers such as ACMO, AMPS, and DMDAAC. The process involves the ultrasonic dispersion of α-LAP, followed by temperature- and pH-controlled polymerization, resulting in a PBMA-co-LAP nanocomposite with enhanced mechanical strength, thermal stability, and rheological properties.

Modern hydrocarbon exploration aims to stabilize wells, limit fluid loss, and inhibit shale from expanding. Adding these nanocomposites to drilling fluid formulae directly helps with all three of these goals. Drilling in deep, unconventional, and extreme settings becomes possible with the specific combination of organic monomers and inorganic nanoparticles, as seen in the image. Figure 3 shows a diagram of how to make PNCs using α-laponite clay nanoparticles [41]. This approach demonstrates how customized interactions between polymers and nanoparticles can yield composites that function more effectively under HPHT conditions for controlling rheology and filtration. It highlights the technological advantages of nanoparticles and polymer matrices, as well as the need for further research to understand and enhance their interaction in real-life downhole scenarios.

#### 2.2.1. PNCs for Improving Rheological Properties

The effectiveness of drilling fluids in various operations, such as cuttings transport, wellbore stability, filter cake formation, and fluid loss prevention, is primarily determined by their rheological properties. Key properties such as yield point (YP), plastic viscosity (PV), apparent viscosity (AV), and gel strength (GS) directly affect a drilling fluid’s ability to suspend and transmit cuttings, generate stable filter cakes, lubricate the drilling bit, and prevent fluid loss into the formation.

In drilling fluids, PNCs are specifically utilized to improve viscosity, yield point, flow behavior, and fluid stability. The high surface area and reactivity of the NPs ensure that the polymer matrix exhibits more interaction, leading to enhanced rheological properties that conventional drilling fluid additives cannot match. The primary function of PNCs in drilling fluids is to improve the rheological properties by introducing NPs such as SiO_2_, TiO_2_, GO, CNTs, and metal oxides into the polymer matrix. These NPs provide a synergistic effect, enhancing the fluid’s viscoelastic properties and enabling it to maintain stability under extreme conditions. By incorporating NPS, PNCs enhance the fluid’s ability to adapt to varying shear rates, ensuring optimal fluid flow and performance during drilling operations.

**Figure 4:**
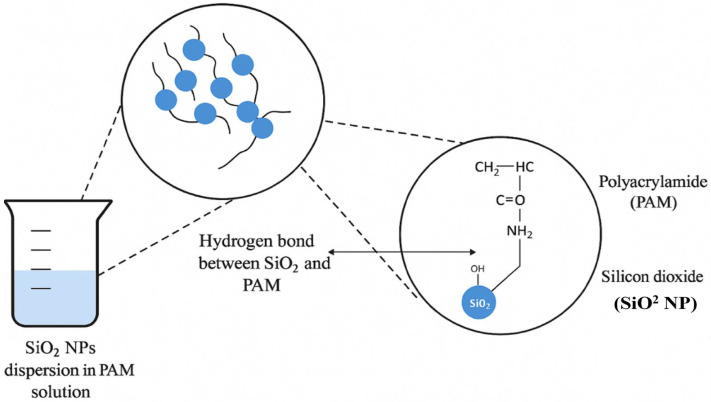
Formation of bonds between PAM and NPs. Reproduced with permission [42]. Copyright 2022, Elsevier.

The mechanism by which PNCs improve the rheological properties of drilling fluids is primarily attributed to the interaction between the polymer matrix and the NPs. NPs help the fluid shear-thin, which is important for drilling. Shear-thinning behavior makes a fluid less viscous as the shear rate increases, making it easier to drill through narrow drill pipes (Figure 4). This property helps PNCs maintain fluid flow while ensuring that cuttings are properly suspended and transported to the surface [42,43]. As a result, PNCs help prevent common drilling issues such as pipe sticking, inadequate cuttings transport, and fluid loss to the formation.

Researchers [44,45,46] examined the use of a titania–polyacrylamide nanocomposite (TiO_2_-PAM NC) in WBMs and observed improvements in the yield point (YP) and plastic viscosity (PV). The addition of TiO_2_-PAM NC also enhanced the shear-thinning rate, meaning the fluid exhibited better adaptability to varying shear rates during the drilling process. As the concentration of TiO_2_-PAM NC increased in the base fluid, the apparent viscosity (AV) decreased, leading to improved fluid flow and enhanced cuttings suspension. Moreover, the addition of TiO_2_ NPs helped form a low-permeability filter cake, which reduced fluid loss to the formation, improving overall wellbore stability.

Similarly, researchers [45] developed a polyacrylamide-grafted polyethylene–silica nanocomposite (PAM-g-PEG SiO_2_ NC) and demonstrated that the PNCs exhibited better rheological properties than pure polyacrylamide (PAM). The PNCs showed increased PV, YP, AV, and GS, which contributed to improved suspension capabilities and better cutting transport. The SiO_2_ NPs enhanced the viscosity of the fluid, allowing it to adapt to varying drilling conditions. Furthermore, the PNCs exhibited improved filtration properties, with the formation of a low-permeability filter cake that reduced fluid loss to the formation [47,48].

**Figure 5:**
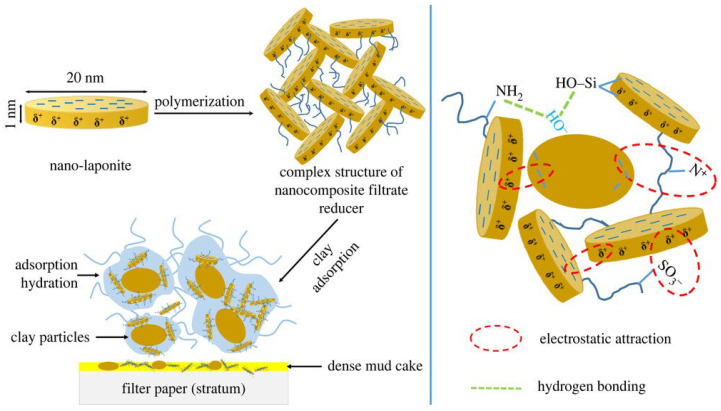
The process by which a polymer nanocomposite may be used to reduce fluid loss [51].

One of the most important challenges for conventional drilling fluids is maintaining rheological stability at high temperatures and pressures. For instance, researchers [49,50] tested a polyethylene–nanosilica composite (PEG-SiO_2_ NC) modified with sodium dodecyl sulfate (SDS) in WBMs at temperatures of 78 °F and 250 °F. The study found that the SDS-modified PNCs significantly improved AV and rheological stability at both temperatures [50]. The SDS-modified PNCs particles interacted with the bentonite clay in the mud, leading to enhanced viscosity and structure stability. Moreover, the filtration properties of the fluid improved, with reduced fluid loss and the formation of a denser, more permeable filter cake. Figure 5 illustrates that utilizing filtrate reducers composed of nano-laponite-based PNCs improves the performance of drilling fluid in challenging drilling situations. Particles of disc-shaped nano-laponite that are roughly 20 nm wide and 1 nm thick [51]. Polymerization transforms these nanoparticles into a complex nanocomposite, which helps reduce the amount of filtrate. When these nanocomposite materials are mixed with the drilling fluid, they react with the clay particles already present in the wellbore. When PNCs stick to the surfaces of clay particles through both hydration and adsorption, as seen in the lower left, they form a dense and compact mud cake at the interface with the geological deposit (filter paper/stratum). This deep mud cake creates a physical barrier that prevents fluids from entering the formation, responding to the urgent need to avoid fluid leakage and stabilize the wellbore. Figure 5 shows how nano-laponite PNCs adhere to clay surfaces and form compact filter cakes, which directly supports their role in preventing fluid loss.

To improve the nanocomposite’s performance, the molecules interact, as seen in the right panel. The polymer matrix and laponite discs influence the clay surfaces through multiple mechanisms, including electrostatic attraction (indicated by the red dashed circles) and hydrogen bonding (represented by the green dashed lines) between functional groups such as NH_2_, HO-Si, and SO_3_^−^. The stable nanoparticles and well-encapsulated clay particles result from the strong interactions that form between their surfaces. In addition to strengthening the mud cake structure, these robust physicochemical interactions prevent the shale from becoming too saturated and elongating, which enhances the thermal stability, mechanical strength, and rheological properties of the drilling fluid [52,53].

Similarly, Researchers [54,55] demonstrated that the inclusion of ZnO-PAM NC in WBMs led to an 18.8% improvement in PV and a 16.7% increase in YP. The ZnO-PAM NC enhanced the rheological properties of the fluid and reduced fluid loss to the formation. These improvements were attributed to the synergistic interactions between the ZnO NPs and the polymer matrix. Although PNCs offer important enhancements in rheological properties, there are still challenges that need to be addressed. One of the primary concerns is the aggregation of NPs within the polymer matrix, which can compromise the effectiveness of PNCs. Aggregation leads to a decrease in the surface area and dispersion of the NPs, affecting the overall rheological performance of the fluid. To overcome this issue, surface modification techniques, such as the use of surfactants and polymers, have been developed to improve the dispersion and stability of the NPs.

Research regularly demonstrates that PNCs improve rheology more efficiently than traditional polymers, especially under HPHT settings. Composites made of silica and graphene exhibit better shear-thinning and viscosity control, whereas systems composed of TiO_2_ and ZnO yield more stable structures. Nanoparticle aggregation remains a problem, and surface modification is often necessary to maintain optimal performance. Future research should focus on modifying techniques that are scalable and economical. Table 3 shows that nanocomposites made of silica and graphene have better rheological properties than conventional polymer additives.

Comparative studies show that PNCs retain viscosity and yield point better than traditional polymer additives under HPHT settings. PNCs composed of silica and graphene have stronger shear-thinning performance, while composites of TiO_2_ and ZnO stabilize structures and prevent fluid leakage. When compared to conventional polymer additives like PAM or PHPA, PNCs consistently demonstrate their ability to withstand higher temperatures and salt. This indicates that they are suitable for deep-sea and unusual drilling situations. However, differences in performance also demonstrate that the choice of nanofiller has an important impact on the balance between rheology, thermal stability, and environmental compatibility.

#### 2.2.2. PNCs for Controlling Filtrate Loss

Filtrate loss is one of the most critical challenges in drilling operations, affecting both wellbore stability and reservoir productivity. The process of filtrate loss occurs when the liquid phase of the drilling fluid seeps into the formation through the pore spaces. This results in the formation of a filter cake on the borehole wall, which acts as a seal, preventing further fluid infiltration. While the formation of the filter cake is a necessary function, excessive filtrate loss can result in thicker filter cakes, increasing the potential for operational issues such as pipe sticking, torque, drag, and the creation of tight holes [84].

The filter cake thickness (FCT) is directly related to the volume of filtrate lost during the drilling process. As more fluid is lost to the formation, the filter cake becomes thicker, and operational challenges become more pronounced. Excessive filtrate loss leads to increased fluid consumption, higher operational costs, and the potential for formation damage. Additionally, high filtrate loss leads to the contamination of formation fluids, which is detrimental to the reservoir’s productivity. It is widely accepted that filtrate loss increases with rising temperature and decreasing viscosity [85,86]. When the viscosity of the fluid decreases, it becomes more challenging to control filtrate loss, especially under extreme conditions like HPHT. To address the challenges associated with filtrate loss, there has been an increasing interest in the development of PNCs as additives in drilling fluids Table 4.

**Figure 6:**
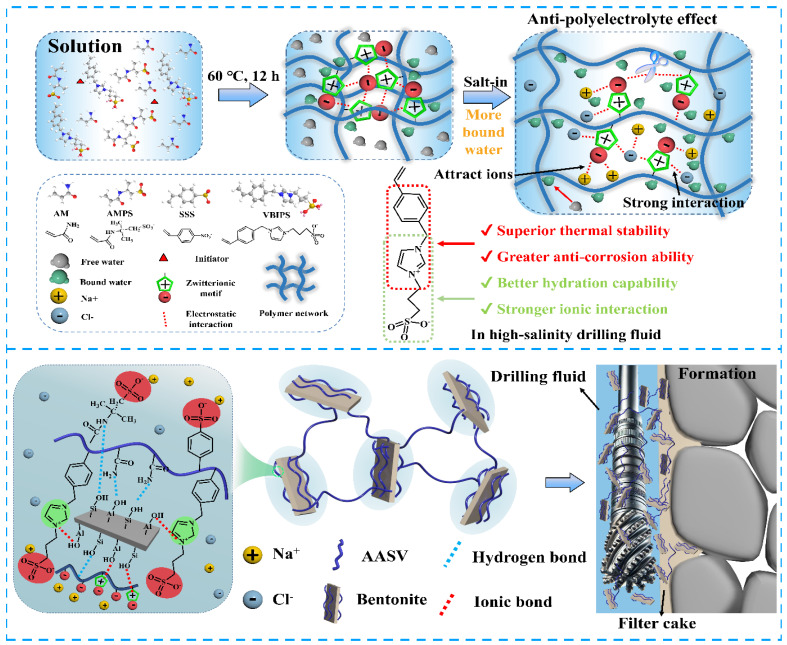
A schematic illustration depicting the zwitterionic copolymer (AASV) synthesis and its mechanism in enhancing the filtration control performance of drilling fluid under high salinity conditions. Reproduced with permission [92]. Copyright 2025, Elsevier.

The large surface area and high surface reactivity of the nanoparticles promote uniform filter cake formation, a critical factor in minimizing filtrate loss. As shown in Figure 6, recent studies have employed advanced molecular design techniques to develop polyzwitterionic PNCs that function effectively as filtration-control additives in water-based drilling fluids under high-salinity and elevated-temperature conditions. The top portion illustrates the process of creating a zwitterionic polymer network (AASV) through the copolymerization of acrylamide (AM), AMPS, sodium styrene sulfonate (SSS), and a phenyl-methylene-imidazole zwitterionic monomer. Researchers synthesized a polymer that shows an important anti-polyelectrolyte effect, unlike regular polyelectrolytes. This polymer stabilizes the colloidal matrix, making it easier for water molecules to bind to it due to its higher ionic strength. Adding the phenyl-methylene-imidazole structure greatly stiffens the polymer backbone and enhances its ability to bind salt, resulting in increased stability at high temperatures [90]. This demonstrates, for instance, that prolonged exposure to high salinity and temperatures considerably reduces HTHP and API filtering losses. These molecular design strategies represent an important shift in the formulation of drilling fluids. They lay the groundwork for the scientifically sound development of next-generation additives tailored to the challenging conditions encountered in modern hydrocarbon exploration and extraction. Figure 6 is aschematic illustration depicting the synthesis and mechanism of zwitterionic copolymer (AASV) in enhancing the filtration control performance of drilling fluid under high-salinity conditions. Reproduced with permission [91]. Figure 6 provides a molecular-level view of how zwitterionic PNCs are designed. It demonstrates how ionic interactions enhance filtration control when salinity levels are high. This supports the idea that chemical design methods are just as important as physical plugging when it comes to preventing fluid loss.

In addition to enhancing filter cake formation, PNCs also improve the rheological properties of the drilling fluid. The polymer matrix in PNCs provides flexibility and adhesion to the drilling fluid, ensuring that the filter cake adheres firmly to the formation surface. The NPs further improve the adhesion properties of the fluid, enhancing the stability of the filter cake and preventing it from being displaced under the pressure exerted by the drilling fluid. This improvement in filter cake adhesion helps prevent the filter cake from breaking apart, which can lead to filtrate infiltration into the formation. By enhancing both the viscosity of the drilling fluid and the adhesion of the filter cake, PNCs help ensure that the filter cake remains stable and effective throughout the drilling process.

A recent study has highlighted the potential of PNCs in controlling filtrate loss and improving filter cake formation. Researcher [92] developed a PNCs additive for bentonite-based WBMs and sodium carbonate-based WBMs. The PNCs were synthesized using acrylamide, AMPS, dimethyl diallyl ammonium chloride (DMDAAC), and modified nano-laponite. The results of this study showed that the PNCs greatly reduced filtrate loss after thermal aging at 150 °C, with the fluid loss of the PNC-modified fluid being 6.4 mL, compared to 10.4 mL for the base bentonite-based fluid. This reduction in filtrate loss was attributed to the colloidal protection provided by the PNCs, which stabilized the clay particle aggregates and formed a denser filter cake that effectively sealed the formation and prevented further fluid penetration. Table 5 shows that nanoclay-based composites reduce the most filtrate, indicating that they could be beneficial in HPHT drilling.

When comparing different types of PNCs, nanoclay- and GO-based ones lose the most fluid at high temperatures. Silica- and polymer-blend composites, on the other hand, show more moderate gains but make the filter cake more stable. The results show that single nanofillers can help limit fluid loss, while hybrid systems (such as PAM–GO and PAM–ZnO) work more effectively to improve both rheology and filtrate control in a balanced manner. Therefore, the integration method, whether it involves hybridization or surface modification, appears to be the most crucial factor in achieving optimal performance from drilling fluid. By creating thin, low-permeability filter cakes, PNCs effectively prevent filtrate loss. Nanoclay- and GO-based systems work best when the temperature is high. Hybrid composites, on the other hand, increase both rheology and filtration in a balanced way. One important problem is that there hasn’t been enough field-scale testing, which highlights the need for pilot studies in genuine drilling operations.

**Figure 7:**
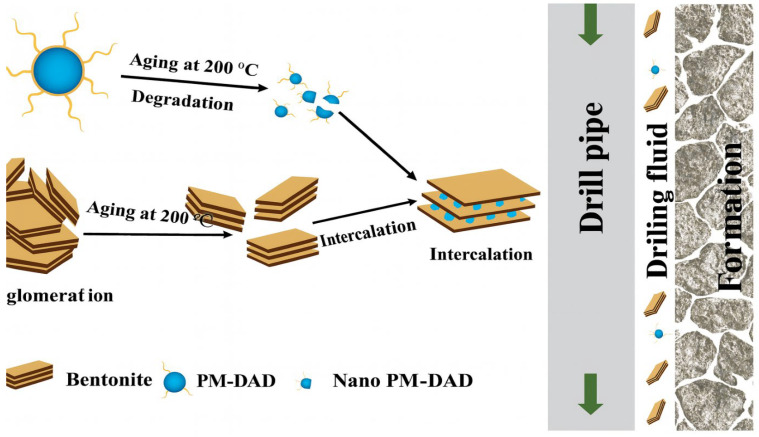
Schematic diagram of ZSHNM filtration control mechanism. Reproduced with permission [107] Copyright 2024, Elsevier.

In another study, researcher [106] synthesized a GO-PAM NC and tested it in WBMs. The results of this study demonstrated that incorporating GO-PAM NC into the drilling fluid improved the rheological properties of the fluid and reduced filtrate loss compared to using GO nanosheets. The study found that the PAM matrix enhanced particle adhesion, while the GO NPs sealed the pore spaces in the filter cake, preventing mud cake erosion and reducing filtrate loss. The incorporation of GO-PAM NC was shown to minimize mud cake erosion by over 60% under dynamic shear conditions, demonstrating the PNC’s effectiveness in improving filter cake stability at high shear rates. Figure 7 shows a diagram of hyperthermal drilling fluids that contain nano-scale polymer-modified drilling additives (Nano PM-DAD). It also demonstrates their stability at high temperatures, their interaction with each other, and their interaction with the wellbore. The diagram illustrates the effects on several parts of the left side when they are exposed to heat at 200 °C for an extended period. Bentonite particles tend to clump together and lose their colloidal stability and performance if they are not appropriately handled. PM-DAD particles, on the other hand, are prone to breaking down when exposed to such high temperatures. The smaller particle size and higher temperature resistance of Nano PM-DAD, on the other hand, allow bentonite platelets to intercalate. Using this intercalation process to make composites makes them less likely to break down or adhere to each other when exposed to heat. This makes them ideal for use in high-temperature environments [95].

Further research by [108] tested the filtrate loss control capabilities of a Sodium Dodecyl Sulfate (SDS)-modified polyethylene–nanosilica composite (PEG-SiO_2_ NC) in WBMs. The study found that the addition of the PNCs resulted in greatly reductions in both filtrate loss and filter cake thickness (FCT). The optimal concentration of the PNCs was found to be 1.5 g, which formed a low-permeability filter cake that effectively sealed the formation and prevented further penetration of the filtrate. The results of this study confirm the ability of PNCs to provide pore-sealing integrity and improve fluid loss control under both API and HPHT conditions, highlighting the importance of PNCs in high-pressure and high-temperature drilling environments (Figure 7).

Another benefit of PNCs is their chemical resistance, which enables them to perform well in environments with high salinity, contamination, or unconventional drilling conditions (Table 6). The high surface area of the NPs enhances the dispersion and stability of the PNCs, ensuring that they remain active and effective throughout the drilling process. This ability to perform well in challenging environments makes PNCs a highly versatile additive for improving fluid loss control in various drilling applications.

#### 2.2.3. PNCs for Inhibiting Shale Swelling

Shale instability due to swelling remains an important challenge in drilling operations. Percentage of shale retained after hydration test: Sensitive clay minerals in shale formations, such as smectite, montmorillonite, and kaolinite, exhibit high hydration potential when exposed to water-based drilling fluids, resulting in rapid volumetric expansion and loss of structural integrity. These minerals have a high affinity for water. When they encounter water-based drilling fluids, they absorb water, causing the shale to swell and potentially leading to operational problems such as pipe sticking, formation damage, and wellbore instability. The problem is particularly severe in tight drilling windows in depleted reservoirs or deep-sea drilling operations, where even minor formation issues can have substantial consequences. The primary mechanism through which PNCs inhibit shale swelling is by increasing the hydrophobicity of the shale and physically blocking pore throats in the shale’s structure. PNCs consist of a polymer matrix and NPs, such as SiO_2_, GO, CNTs, and CNCs [23]. These NPs are highly reactive and possess an important surface area, allowing them to interact directly with the shale’s surface, reduce water absorption, and prevent the shale from swelling. When PNCs are added to the drilling fluid, they adhere to the surface of the shale, forming a protective layer that prevents water from penetrating the shale and causing it to swell.

##### Shale Swelling Mechanisms and Mineralogical Basis

The hydration and swelling of clay minerals, such as smectite and montmorillonite, primarily cause the instability of shale. These minerals have a layered structure with voids between the layers that can hold water. These clays have a high cation exchange capacity (CEC), which means that interlayer cations (Na^+^, K^+^, Ca^2+^) can swap places with ions in the drilling fluid. This causes the volume to grow. When polar water molecules get between the layers, they break down the electrostatic interaction between the sheets and push them apart. An osmotic imbalance between the clay pore fluid and the external drilling mud also causes water to flow in, exacerbating the swelling. The effects of surface charge are also important, clay platelets with a negative charge attract hydration shells and more cations, which makes dispersion and structural weakening worse. WBMs do not restrict shale formations from being water-sensitive, while PNCs physically block nanopores and produce hydrophobic barriers to prevent hydration, osmotic, and ion exchange. This makes them a stronger and more chemically compatible solution for formations that are prone to shale.

Different types of nanoparticles interact with shale minerals in various ways. Silica nanoparticles (SiO_2_) mainly stop shale from getting wet by neutralizing the charges on the surface. Their negatively charged surfaces can interact with the diffuse double layer that surrounds clay platelets. This compresses the layer and lowers the repulsive forces that cause interlayer swelling. GO renders clay surfaces hydrophobic due to its amphiphilic nature. This makes it harder for water to stick to the surface by forming a barrier layer that prevents hydration shells from penetrating the interlayers. Carbon nanotubes (CNTs) have a high aspect ratio and a tubular shape, which enables them to bridge. This makes it harder for water to get in. These mechanisms work together to improve the polymer matrix: the polymer adheres to the nanoparticle surface, facilitating its adhesion, while the nanoparticles activate the prevention of hydration, ion exchange, and fluid invasion. This synergy is why PNCs work better than single-component additions in strata that are prone to shale.

**Figure 8:**
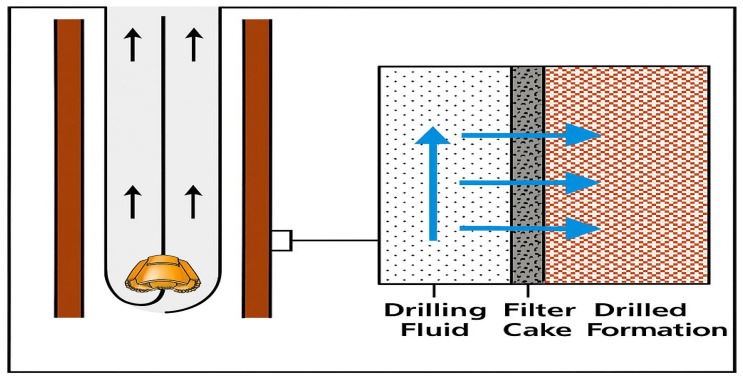
Filter cake formation in drilled formations by drilling fluid [23].

By placing all the shale-related failures (instability, pipe sticking, collapse) here, rather than in the introduction, the discussion clarifies that the hydration process directly causes these operational problems. The PNCs that are offered (Figure 8) show how filter cakes form in shale strata that have been treated with PNCs. This diagram is closely related to the conversation about how to prevent shale from expanding, as it illustrates how interactions between nanoparticles and polymers block pore mouths and maintain formation stability.

Among the various nanoparticles utilized for stabilizing bentonite in high-temperature drilling fluids, nano-silica (SiO_2_ nanoparticles) is particularly notable. When incorporated as a polymer-modified additive, nano-silica not only resists thermal degradation but also efficiently intercalates between bentonite layers, maintaining colloidal stability and improving filtration control in challenging downhole environments. Additionally, the polymer matrix in PNCs serves to stabilize the NPs, ensuring they remain evenly dispersed in the drilling fluid. This ensures that the PNCs can continue to adhere to the shale surface and provide effective shale stabilization over time. By stabilizing the NPs, the polymer matrix also helps prevent nanoparticle aggregation, which could reduce the effectiveness of the PNCs in stabilizing the shale.

Recent studies have demonstrated the efficacy of PNCs in inhibiting shale swelling and stabilizing shale formations during drilling operations. Resercher [112] developed a PNCs additive for bentonite-based WBMs and sodium carbonate-based WBMs. The PNCs were synthesized using acrylamide, AMPS, dimethyl diallyl ammonium chloride (DMDAAC), and modified nano-laponite. The study revealed that the addition of PNCs improved the fluid retention and reduced the filtrate loss. The PNCs enhanced the structural stability of the shale by reducing shale swelling and mud cake erosion. When tested at 150 °C, the PNCs reduced the filtrate loss from 10.4 mL to 6.4 mL, demonstrating the effectiveness of PNCs in stabilizing shale and reducing filtrate loss in high-temperature environments.

Another promising study by [113] focused on a Sodium Dodecyl Sulfate (SDS)-modified polyethylene–nanosilica composite (PEG-SiO_2_ NC). The results showed that these PNCs reduced filtrate loss and filter cake thickness (FCT), while improving the percentage of shale retained after the hydration test. The PNCs formed a low-permeability filter cake that effectively sealed the formation and prevented further penetration of the filtrate. The study indicated that the PNCs enhanced the hydration resistance of the shale and improved the overall shale stabilization in WBMs. This study further corroborates the role of PNCs in shale stabilization and swelling inhibition during drilling operations [114].

**Figure 9:**
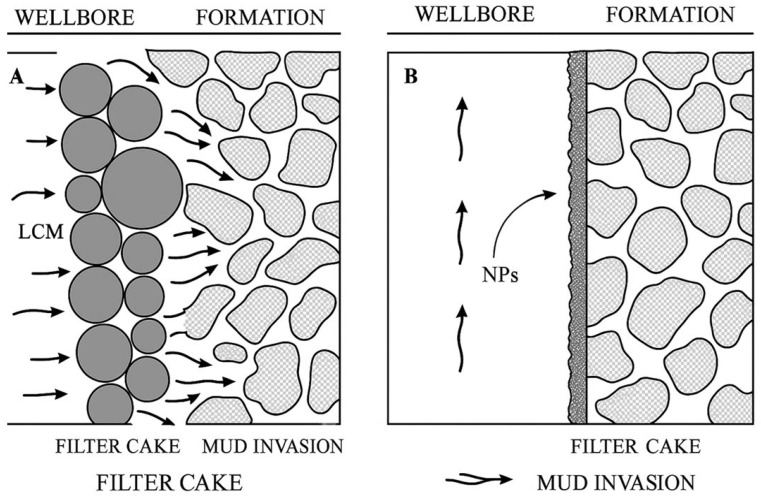
A schematic illustration of mud losses during drilling in the scenarios of (**A**) conventional Lost Circulation Material (LCM) and (**B**) Non-Newtonian Polymer (NP). Reproduced with permission [7] (Copyright 2015, Springer Nature).

Hydrophobicity is a critical factor in shale swelling inhibition. Hydrophobic NPs such as SWCNTs can reduce shale swelling by repelling water and preventing water absorption into the shale matrix. In addition to hydrophobicity, PNCs also act through physical blocking mechanisms. NPs in the PNCs can physically block the pores and microcracks in the shale, preventing water from entering the shale structure. SWCNTs/PVP (single-walled carbon nanotubes and Polyvinylpyrrolidone) nanocomposites, for instance, exhibit a higher ability to stabilize shale by adsorbing onto the shale surface and blocking microcracks. FTIR analysis of SWCNTs/PVP-treated shale showed too much reduction in the -OH stretch peak, indicating decreased water adsorption and increased hydrophobicity (Figure 9). These findings suggest that PNCs with hydrophobic properties can effectively reduce shale swelling and enhance shale stability in water-based drilling fluids. Table 7 shows that graphene-based nanocomposites always work better than silica-based systems at stopping shale from swelling because they are hydrophobic.

##### Mechanism of Shale Swelling

Shale formations, which contain an important proportion of clay minerals, are prone to swelling when exposed to water-based drilling fluids (WBDFs). The primary mechanism driving this swelling is the interaction between water and the clay minerals, especially montmorillonite, which is highly hydrophilic. These clay minerals have a layered structure that contains cations (such as sodium, calcium, or potassium) in the interlayer spaces. When exposed to water, these cations are hydrated, causing an increase in the distance between clay layers, a process known as crystalline swelling.

There are two main types of swelling mechanisms in clay minerals: crystalline swelling and osmotic swelling. In crystalline swelling, water molecules form layers around the cations in the interlayer spaces, increasing basal spacing. This expansion is typically limited to a few layers of water due to electrostatic repulsion between the layers. Osmotic swelling occurs when there is a difference in ionic concentration between the interlayer cations and the surrounding water. This leads to the diffusion of water molecules into the interlayer space to balance the ion concentration, greatly increasing the swelling pressure between the layers.

The swelling of shale can have severe consequences for wellbore stability during drilling operations. As the shale absorbs water, the volume of the clay minerals increases, causing mechanical failure in the wellbore wall. This can lead to borehole collapse, sloughing, or excessive enlargement of the hole, all of which complicate drilling operations. Furthermore, the dispersion of fines from the swollen shale can contaminate the drilling fluid, degrade its performance and increase the risk of lost circulation or stuck pipe incidents.

To mitigate the adverse effects of shale swelling, chemical inhibitors are incorporated into drilling fluids. These inhibitors work by modifying the interaction between water and clay minerals, either by encapsulating clay particles to prevent water absorption or by neutralizing the surface charge of the minerals to reduce their tendency to swell. Advanced inhibitors, such as ionic liquids (ILs) and deep eutectic solvents, offer enhanced inhibition capabilities. These compounds function through hydrogen bonding, electrostatic attraction, and hydrophobic shielding, providing a more effective barrier against water penetration and reducing swelling pressures.

Cationic organic polymers (COPs) notably quaternary polyamines and quaternary polyacrylamides adsorb multipoint onto negatively charged clays, immobilizing PNCs and forming a barrier to water ingress. Hyperbranched, amine-terminated designs enhance binding via protonated primary amines, suppressing Na-bentonite hydration and dispersion. Their cationic nitrogen anchor to clay while hydrophobic backbones create a network that limits fines migration and reduces water uptake, outperforming single-cation inhibitors in WBDFs.

Graphene- and CNT-based PNCs are very good at stopping shale from expanding because they repel water and plug pores. Silica-based PNCs are cheap, but they don’t work as well. Hybrid systems that mix polymers with oxide or carbon fillers are more stable. Even though the lab results were good, there is still some doubt about how stable the system will be in the long run when conditions change downhole.

PNCs offer a promising solution for inhibiting shale swelling and stabilizing shale formations during drilling operations. By incorporating NPs such as SiO_2_, GO, CNTs, and CNCs into a polymer matrix, PNCs improve the hydrophobicity of the shale and reduce water absorption, thereby preventing shale swelling. The ability of PNCs to block pores, seal microcracks, and increase shale stability makes them a valuable additive in water-based drilling fluids. As drilling conditions become increasingly challenging, the use of PNCs will continue to play a crucial role in improving shale stabilization, reducing filtrate loss, and enhancing the overall performance of drilling fluids. Future research and development of PNC formulations will continue to provide more effective and cost-efficient solutions for shale swelling inhibition and wellbore stabilization.

Comparative analyses indicate that graphene- and CNT-based nanocomposites exhibit the most effective long-term suppression of shale swelling, attributed to their hydrophobic characteristics and pore-blocking capabilities. Silica-based composites, on the other hand, provide moderate inhibition but are more chemically stable and less expensive. Hybrid PNCs, such as ZnO or GO–PAM systems, achieve both objectives: they enhance shale stability while maintaining control over filtration. This comparative research supports the perspective that hybrid nanocomposites represent the most advantageous pathway for practical field applications.

**Table 7:** Current analysis of PNCs materials utilized as shale swelling inhibitors and viscosifiers in WBM drilling, including thermal stability thresholds and quantitative swelling inhibition metrics.

Material Used (PNCs)	Polymer Used	Nanofillers Used	Thermal Stability Threshold (°C)	Swelling Inhibition/Shale Recovery Metrics	Key Findings	Shale Inhibition Mechanism	Reference
PEI–Gr NC	Polyethyleneimine (PEI)	Graphene (Gr)	180 °C	Shale recovery 65%; swelling reduction ≈ 45% vs. base mud	Maintained stability under HPHT; enhanced hydrophobic sealing	PEI interacts with –OH groups on the shale surface; Gr creates a barrier layer blocking microcracks	[115]
Glu–Gr NC	Glutamic acid (Glu)	Graphene (Gr)	200 °C	Swelling reduction ≈ 52%; shale recovery 70%	Long-term shale stability after 48 h aging at 150 °C	Hydrogen bonding binds Gr to shale; intercalation prevents water penetration into nanopores	[116]
PNS Latex	Polymer (PNS)	Latex NPs	160 °C	API FL reduced from 24 mL to 10.8 mL; shale recovery ≈ 60%	Greater thermal resistance and filtrate control under HPHT conditions	Adsorbs on shale microstructures, plugging cracks and pores	[117]
PAM–SiO_2_ NC	Polyacrylamide (PAM)	SiO_2_	210 °C	Swelling inhibition ≈ 70%; recovery 86.6% over 48 h	Outperformed commercial inhibitors (KCl 49.2%, base mud 74.7%)	Physical blocking of nanopores and hydrogen bond disruption by SiO_2_–OH groups	[118]
C-g-AA–NH_2_ NC	Acrylic acid (AA)	Aminomethyl, Amyl	190 °C	Anti-swelling ratio 95.2%; recovery > 80%	Effective plugging and surface adsorption at moderate HPHT	Carbon-core nanopore plugging and strong polymer–shale adsorption	[119]
AP–ZnO NC	Acrylic Polymer (AP)	ZnO	220 °C	Swelling rate reduced from 14.3% to 6.7%; recovery 97%	Good thermal endurance and inhibition under saline conditions	ZnO plugs pores and cracks; AP encapsulates the shale surface and reduces water contact	[120]
GO–PAM NC	Polyacrylamide (PAM)	GO	230 °C	Swelling reduction ≈ 60%; shale recovery 90%	Retained integrity after 150 °C aging; thin filter cake formation	GO sheets provide a hydrophobic barrier and improve shale cohesion	[121]
SWCNTs/PVP NC	Polyvinylpyrrolidone (PVP)	SWCNTs	240 °C	Swelling reduction ≈ 68%; FTIR shows reduced –OH absorption	Outstanding shear and thermal resilience under HPHT conditions	Hydrophobic shield and micro-pore plugging mechanism	[122]

#### 2.2.4. PNCs for Stabilizing the Wellbore

Wellbore instability is a common and costly issue encountered during drilling operations, especially in challenging environments such as deep-sea drilling or depleted reservoirs. The primary cause of wellbore instability is the reaction between water-sensitive clay minerals in shale formations and the drilling fluids, which leads to problems like sloughing, pipe sticking, formation damage, and wellbore collapse. These issues can result in the loss of circulation, poor wellbore cleaning, and, in extreme cases, formation failure. Therefore, stabilizing the wellbore is crucial to ensure the integrity of the drilling operation. To address these challenges, PNCs have emerged as effective additives in drilling fluids, offering solutions for wellbore stabilization. Figure 9 illustrates how traditional loss circulation materials (LCMs) and nanoparticle-based filter cakes effectively seal and filter drilling fluids. At the contact between the wellbore and the formation, conventional LCMs typically form a rough filter cake with large, irregular particles. This filter cake allows a good amount of muck to enter the formation’s porous structure due to the relatively large particles, which also result in poor sealing. Hydrocarbon exploration is inherently risky, especially in complex conditions were insufficient plugging can facilitate fluid escape and reduce wellbore stability. Figure 9 shows the utilization of NPs in the drilling fluid system, including nano-silica (SiO_2_ NPs), nano-titania (TiO_2_ NPs), and GO nanosheets. The high surface area and diminutive size of these NPs allow them to penetrate even the most microscopic pore spaces found in the formation. The final product is a filter cake with reduced mud invasion, thanks to its thinner, more compact, and less porous structure. This nanoscale sealing improves wellbore stability, decreases fluid loss, and is most effective in environments with high salt, heat, and pressure. This illustration demonstrates that utilizing drilling fluids reinforced with nanoparticles can enhance well control and operational safety in contemporary hydrocarbon exploration. Figure 9 shows the difference between traditional loss circulation materials and nanoparticle-based PNCs. It demonstrates why nanoscale sealing results in cakes that are thinner and less permeable, thereby making the wellbore more stable.

**Figure 10:**
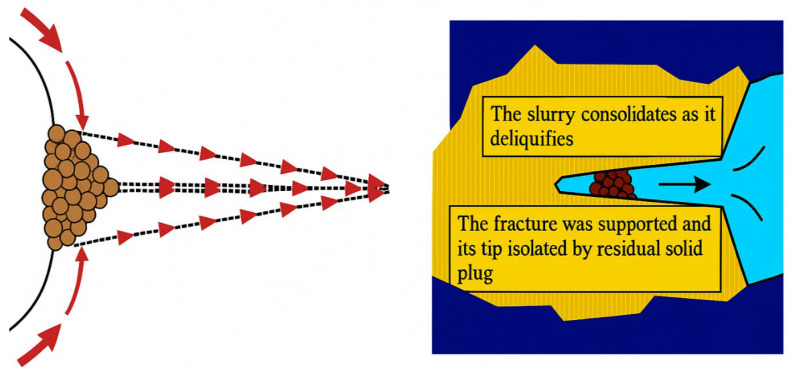
Wellbore stabilizers may separate the wellbore from the fracture at the penetration. Reproduced with permission [124] or permit the fracture to enter the wellbore.

Several studies have demonstrated the efficacy of PNCs in stabilizing wellbores during drilling operations. Dong et al. [123] developed a PNC additive for bentonite-based WBMs and sodium carbonate-based WBMs. The PNCs were synthesized using acrylamide, AMPS, dimethyl diallyl ammonium chloride (DMDAAC), and modified nano-laponite. The study demonstrated that the PNCs enhanced wellbore stability by improving the structural integrity of the shale and forming a dense filter cake that prevented the infiltration of drilling fluid into the formation. After thermal aging at 150 °C, the PNCs reduced the filtrate loss from 10.4 mL to 6.4 mL, demonstrating their effectiveness in stabilizing the wellbore under high-temperature conditions. Figure 10 highlights wellbore stabilization strategies with PNCs, showing fracture sealing at the penetration point.

In a similar study, researcher [125] synthesized a GO-PAM NC and tested its wellbore stabilization properties. The results showed that incorporating GO-PAM NC into the drilling fluid significantly improved the rheological properties of the fluid and enhanced wellbore stability. The GO-NPs helped seal the pores in the shale, preventing water penetration and swelling. At the same time, the polyacrylamide (PAM) matrix increased the adhesion between the NPs and the shale surface. This study indicated that PNCs effectively stabilize the wellbore by creating a mechanical barrier that prevents the drilling fluid from infiltrating the shale and protects the formation from damage.

The hydrophobicity of PNCs plays a key role in stabilizing the wellbore, particularly in formations with water-sensitive shale. Hydrophobic NPs such as SWCNTs can reduce shale swelling by preventing water from entering the shale. For instance, the SWCNTs/PVP (Polyvinylpyrrolidone) nanocomposite has demonstrated decent performance in wellbore stabilization. Fourier-Transform Infrared Spectroscopy (FTIR) and Field-Emission Scanning Electron Microscopy (FESEM) analysis showed that the SWCNTs/PVP nanocomposite reduced the -OH stretch peak in the FTIR spectra, indicating a decrease in water adsorption and an increase in the hydrophobicity of the shale surface (Figure 10). This increase in hydrophobicity helps to reduce shale swelling and maintain wellbore stability by preventing the shale from absorbing water, which would otherwise lead to wellbore instability [126,127].

In addition to hydrophobicity, PNCs also prevent wellbore instability through physical blocking mechanisms. NPs in the PNCs can block the pores and microcracks in the shale, physically preventing water from penetrating the shale and causing it to swell. For example, PNSC + NH_2_ (Polymer Nanocomposite with Amine Functional Groups) has been shown to effectively plug pores in the shale and prevent water absorption. The hydrophobic shield created by PNCs + NH_2_ reduces shale swelling and enhances wellbore stability by limiting water interaction with the shale.

PNCs offer several advantages over traditional wellbore stabilizers in drilling fluids. The dense filter cake formed by PNCs prevents fluid loss and protects the formation from damage. By sealing microcracks and pore throats in the shale, PNCs reduce the risk of shale swelling, formation damage, and wellbore collapse. The NPs in PNCs enhance the mechanical strength of the drilling fluid, making it more effective in stabilizing the wellbore under extreme conditions, such as HPHT environments. PNCs also improve the rheological properties of the fluid, ensuring that it maintains its flow characteristics and particle suspension capabilities even under challenging conditions.

**Figure 11:**
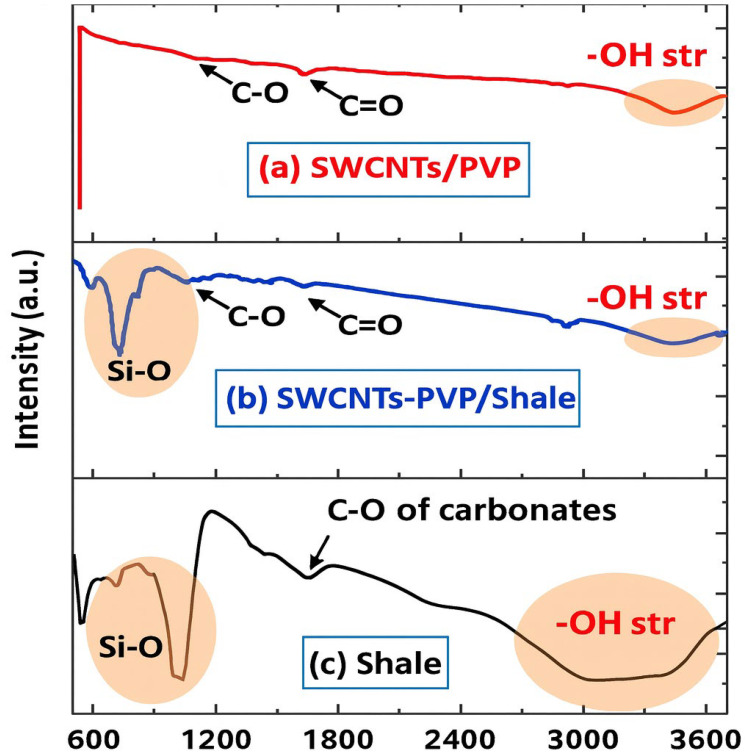
FTIR spectra of (**a**) SWCNTs/PVP nanocomposite, (**b**) SWCNTs/PVP nanocomposite changed with shale, and (**c**) shale pellet that has not been treated. Reproduced with permission [128]. Copyright 2020, Elsevier.

Additionally, PNCs can be customized to fit specific wellbore conditions, including shale type, temperature, and salinity. The polymer matrix in PNCs allows for modification of the fluid’s viscosity, yield point, and filtration properties to suit the specific needs of the drilling operation. This versatility makes PNCs an ideal solution for wellbore stabilization in various drilling environments. Researchers increasingly utilize PNCs in drilling fluids to stabilize shale during wellbore development. These nanocomposites block pore throats, generate internal filter cakes, and reduce fluid incursion, lowering wellbore shale permeability. PNCs increase shale’s hydrophobicity, limiting cation exchange with WBMs, which causes expansion and instability. PNCs can intercalate in the shale’s clay matrix, reducing its expansion and increasing structural stability [46]. A nanocomposite of SWCNTs and PVP was produced and named SWCNTs/PVP NC. They examined the nanocomposite’s swelling inhibition mechanism and interaction with the shale core using FESEM and FTIR. In FTIR, SWCNTs/PVP-treated shale had a greatly lower -OH stretch peak region than untreated shale (Figure 11). The brown peak indicates decreased water adsorption and higher hydrophobicity. However, the untreated shale pellet had a larger -OH stretch peak, indicating hydrophilia. These findings demonstrate the potential of SWCNTs/PVP to stabilize shale and recommend its application in semi-hydrophobic drilling fluids. Figure 11 confirms through FTIR analysis that SWCNTs/PVP nanocomposites reduce water adsorption on shale, supporting hydrophobic stabilization mechanisms.

PNCs offer a highly effective solution for wellbore stabilization during drilling operations. By combining polymers with NPs, PNCs provide enhanced mechanical strength, thermal stability, and chemical resistance. The hydrophobic properties and pore-blocking mechanisms of PNCs help prevent shale swelling and maintain wellbore integrity. In contrast, their filter cake-forming ability prevents fluid loss and protects the formation from damage.

Another issue is the shear resistance of polymer nanocomposites, as drilling fluids undergo high shear forces near the drill bit. If these forces shatter nanocomposites, their efficiency may decrease. Researchers are developing shear-resistant nanocomposites that can withstand tremendous shear stress without degrading. To ensure drilling success, nanocomposites can be made more shear-resistant.

**Figure 12:**
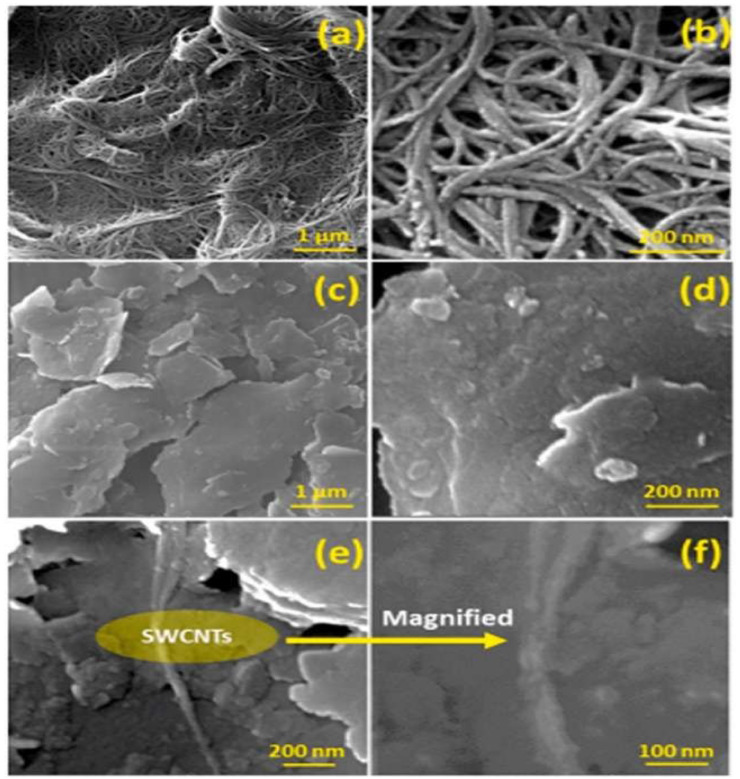
Field emission scanning electron micrographs of (**a**,**b**) pure SWCNTs, (**c**,**d**) untreated shale sample, and (**e**,**f**) SWCNTs/PVP with treated shale. Reproduced with permission [128]. Copyright 2020, Elsevier.

Despite these challenges, PNCs can improve wellbore stabilization. As drilling processes become more complex, nanocomposites will be essential for efficient and reliable drilling. Future studies should focus on addressing cost, environmental impact, and shear resistance to maximize the potential of these materials. Nanocomposite technology enhances performance, affordability, and environmental sustainability, making wellbore stabilization a promising prospect. The structural examination of shale samples treated with SWCNTs/PVP nanocomposites and their effects on the surface characteristics of the shale. Figure 12 illustrates that the imaging approach used for powder sample preparation on a copper stub, devoid of sonication, elucidates the occurrence of SWCNT agglomerates [106,129,130]. The behavior of carbon nanotubes, including van der Waals forces and dipole–dipole interactions, may explain these agglomerates at high magnification. Figure 12 shows SWCNTs as a coherent network on the surface. After disintegrating in water, untreated shale surfaces are rough and porous, with minute particles visible at low resolution. The shale’s holes indicate its vulnerability to water absorption. The SWCNTs/PVP nanocomposite’s protective layer makes the shale’s surface smooth and less porous, minimizing water exposure. This change decreases swelling and dispersion, stabilizing the WBMs shale. The experimental data in Figure 12 show that SWCNTs/PVP NC efficiently obstruct nano-micro-pores, improving shale water resistance and structural integrity during drilling. Figure 12 illustrates, via FESEM, how SWCNT/PVP nanocomposites form a protective layer over shale, thereby reducing porosity and enhancing structural stability.

In a similar work, researcher [128] examined the use of modified polyvinyl alcohol (PVA) nanocomposites to enhance shale stability in WBMs. The findings indicated that incorporating a 1.5 w/v% PVA-based nanocomposite markedly improved the inhibitory characteristics of the base fluid. The swelling index of shale decreased by almost 85% after 24 h of contact with the nanocomposite-amended drilling fluid, in contrast to the base fluid, which exhibited an elevated swelling rate. The PVA-based nanocomposite was observed to create a protective layer on the shale surface, restricting the infiltration of water molecules [129]. The barrier formation primarily occurred due to hydrogen bonding between the hydroxyl (-OH) groups on the shale and the PVA chains, which reduced water absorption on the shale particles. This interaction reduced the swelling and diffusion of clay particles in the wellbore.

Besides, at a high temperature (250 °F), the stability of the PVA nanocomposite was maintained, proving its efficacy in adverse conditions. The addition of the nanocomposite did not alter the rheological properties of the drilling fluid, as indicated by the marginal increase in plastic viscosity (PV) and yield point (YP). The results are potent evidence for the use of PVA nanocomposites as a viable shale improvement in WBMs. The research demonstrated that the nanocomposite’s protective layer can reduce fluid loss and improve the overall efficacy of the drilling mud under challenging conditions.

Both hydrophobic PNCs (like CNT- and graphene-based ones) and oxide-based systems (like silica and titania) can help stabilize wellbores. Oxides make things more stable thermally and structurally, while hydrophobic nanocomposites prevent swelling. The choice depends on the type of formation and the drilling conditions. Cost, environmental problems, and shear resistance are still issues that need to be solved before widespread use can happen.

The effectiveness of AA-AAm-C-Amyl composites, which consist of acrylic polymer and amyl ester-activated carbon, as shale inhibitors. Study shows that the wellbore was successfully stabilized by both the somewhat hydrophobic AA-AAm-C-Amyl composite (with shale recovery capacities of 95.2% and 97%) and the very hydrophobic AA-AAm-OD-C-Amyl composite (with a 93.7% anti-swelling ratio). The composites were evaluated against KCl, a widely used shale swelling inhibitor, and showed bigger efficacy in mitigating shale hydration. The hydrophobic characteristics of the AA-AAm-OD-C-Amyl composite enhanced its effectiveness in reducing shale swelling. However, the C-g-AA-NH_2_ showed inhibitory effects on Na-Bt clay, resembling the smectite constituents of shale formations. These results indicate the capability of these composites to improve drilling fluid efficacy.

Researchers created a PEG-NS composite with dimensions ranging from 110 to 434 nm, which is resistant to high temperatures [130]. They showed that it worked well as a stabilizing agent for shale in traditional WBMs. The composite was tested using rolling dispersion and pore pressure transmission studies. The results showed that PEG-NS limits the transmission of pore pressure and reduces the permeability of shale. Compared to polymeric alcohol (JHC) and KCl, the PEG-NS-based WBMs have effectively alleviated shale swelling at similar concentrations, thus serving as an effective stabilizer and improving the functions of conventional drilling fluids in shale formations.

Researchers [33] investigated how nano-polymer drilling fluids mitigate shale-related problems, focusing on the impact of additives on contact angles and shale expansion. The researchers assessed four different base fluid formulations. BF, BN_4_, BP_4_, and BPN are all formed by combinations of NPs or polymers. The mixture consisting of ZnO-NPs and associative polymer (AP) (BPN_4_) had a maximum contact angle of 64.80°. The increase in contact angle represents the enhanced hydrophobicity of the shale surface, as the nano-polymer composite alters its surface properties. The decrease in shale swelling, from 14.29% using conventional WBMs to 6.69% with the PNCs recipe (BPN_4_), was reported in the research. The findings confirm the ability to incorporate ZnO nanoparticles and ammonium phosphate into drilling fluids to improve shale inhibition and minimize formation damage, which is essential for delivering effective drilling operations.

PNCs in drilling fluids demonstrate an important potential in reducing wellbore instability, particularly in shale accumulations [128]. These fluids are formulated to create a low-permeability filter cake that prevents direct contact between the drilling fluid and the shale, thereby avoiding hydration, swelling, and destabilization risks. Moreover, hydrophobic properties are typically enhanced in PNC-based drilling formulations, thereby reducing the shale’s ability to absorb water from the fluid. The increased hydrophobicity reduces the cation exchange ability of the shale, thus further hindering harmful interactions. As a result, the stability of the wellbore is enhanced, and PNCs become highly effective in controlling the damage caused by fluids in reactive shale formations, thereby improving overall drilling results and efficiency.

PNCs in drilling fluids have the potential to alleviate problems like shale formation stabilization, enhanced rheology, and improved management of filtrate loss. However, some challenges still hinder their full implementation in drilling processes. The uneven distribution of NPs in the polymer matrix causes the fluids to exhibit varying properties and perform poorly. The uneven spread of NPs often weakens the stability of the drilling fluid, affecting its flow and filtration properties. The performance of PNCs depends on NP dispersion. High-pressure homogenization, magnetic stirrers, ball mills, and ultrasonic baths have been used to disperse NPs. Overall, nanocarbon-based composites like graphene oxide and carbon nanotubes create stronger hydrophobic barriers; however, inorganic fillers like silica or titania provide better thermal and structural stability. Therefore, the PNC system you choose should be tailored to the specific circumstances in the wellbore. For example, use hydrophobic nanocomposites for formations that are rich in shale and sensitive to water, and oxide-based nanocomposites for HPHT settings. This framework for comparison facilitates the selection of PNCs for drilling operations in specific fields.

## 3. Conclusions and Future Prospects

Polymer nanocomposites (PNCs) represent an important advancement in drilling fluid technology. They exhibit better rheology, reduced filtrate loss, enhanced shale inhibition, and improved thermal and salt tolerance compared to typical polymer-based additives. Their ability to stabilize wellbores and enhance environmental performance shows that they could be used in the next generation of high-performance water-based drilling fluids (WBDFs).

However, to make this happen, both design and field applications must incorporate sustainability, safety, and predictive performance assessment. Life Cycle Assessment (LCA) and circular economy principles are essential tools for determining the environmental impact of PNCs, from sourcing raw materials to disposal or reuse. There aren’t many LCA studies on drilling fluids currently available, and those that do exist often employ different methods. This makes it hard to compare sustainability. It is suggested that subsequent studies utilize standardized frameworks, such as ISO 14040/14044 and the EU Product Environmental Footprint (PEF) criteria, to facilitate uniform environmental effect assessments across investigations [131].

To ensure that PNC development aligns with the goals of a circular economy, we need to transition toward biodegradable and waste-derived polymers. Lignin-based polymers, which come from pulping or biomass waste, and cellulose nanocrystals (CNCs), which come from agricultural or forestry waste, are two promising candidates. Lignin-based polymers excel at dispersing and altering rheology, while CNCs are strong, functional on the surface, and biodegradable. These bio-based fillers can replace some synthetic monomers, reduce carbon intensity, and make the product easier to break down at the end of its life, all without compromising its thermal or filtering performance. Starch, chitosan, and modified guar derivatives have also been shown to be very beneficial for the environment and may be used on a large scale in WBDFs.

At the same time, the fact that synthetic nanocomposites are harmful and persist in the environment for an extended period remains an important concern. If polyacrylamide-based systems aren’t completely polymerized, residual acrylamide monomers can be detrimental to the environment. Nanoparticle leaching (such as SiO_2_, ZnO, or graphene oxide, or GO) can also lead to bioaccumulation or groundwater contamination. Therefore, operational protocols should include strict control of synthesis conditions, comprehensive verification of polymerization, and proper treatment of drilling fluids after use. Additionally, eco-toxicological tests and assessments of the potential for nanomaterials to leach out should be standard reporting criteria for PNC-based fluid formulations.

The next phase of research should focus on developing multiscale modeling and simulation tools that can accurately replicate PNC behavior in dynamic downhole conditions, thereby enhancing the predictability of field performance. Coupled computational fluid dynamics (CFD) and molecular dynamics (MD) simulations can describe how nanoparticles interact with clay minerals, temperature fluctuations, and salinity fluctuations, providing quantitative insights into rheology and filtration mechanisms before field deployment. Predictive models based on machine learning could also accelerate the development of PNCs by linking data on composition from the lab to performance results in the field.

In short, to improve PNCs for drilling fluids, we need a framework that combines high-performance engineering, long-term sustainability, and predictive modeling. Adding biodegradable, waste-derived polymers, such as lignin, cellulose nanocrystals, and chitosan, to nanocomposite matrices can help protect the environment and promote the goals of a circular economy. Addressing toxicity risks through standardized LCA evaluation and nanoparticle leaching control, alongside the development of data-driven predictive tools, will be pivotal for the sustainable deployment of PNC-based drilling fluids in future hydrocarbon exploration and geothermal applications.

## Figures and Tables

**Table 1:** Key polymeric additives relevant to water-based drilling fluids (WBDFs) and their functional performance.

Polymer	Primary Functions in WBDFs	Advantages	Limitations/Challenges
Xanthan Gum (XG)	Viscosifier, suspension aid, improves gel strength	Exceptional rheology control at low shear rates; eco-friendly and biodegradable	Thermal degradation above 150 °C; viscosity loss in high-salinity environments
Polyanionic Cellulose (PAC)	Fluid-loss reducer, filtrate-control agent	Maintains stable filter cake; good salt tolerance; improves wellbore stability	Sensitive to Ca^2+^/Mg^2+^ contamination; limited performance above 160 °C
Carboxymethyl Cellulose (CMC)	Filtrate control and rheology modifier	Biodegradable; forms thin, low-permeability filter cakes	Degrades above 150 °C; reduced efficiency in highly saline systems
Partially Hydrolyzed Polyacrylamide (PHPA)	Shale encapsulation, viscosity enhancement	Provides very good cutting suspension and shale inhibition	Decomposes under HPHT; limited solids tolerance
Hydrolyzed Polyacrylamide (HPAM)	Rheology enhancer and clay stabilizer	High viscosity at low dosage; improves drilling rate	Mechanically unstable under shear; acrylamide monomer toxicity concerns
Polyanionic Starch (PAS) / Modified Starch	Filtrate-loss control and wellbore stabilization	Cost-effective; biodegradable; good salt tolerance	Loses structure above 180 °C without crosslinking or nanofiller support
Guar Gum (GG)	Viscosifier and lubricity enhancer	Provides high viscosity at low concentrations; bio-based and renewable	Poor thermal stability (>150 °C); bacterial degradation risk
Scleroglucan/Welan Gum	Rheology modifier for HPHT wells	High shear and thermal resistance; stable gel formation	Costly; susceptible to oxidation in long-term storage
Chitosan Derivatives	Rheology and fluid-loss modifier	Biodegradable; enhances thixotropy and film-forming capability	Thermal degradation above 160 °C; limited salt tolerance
Potassium Polyacrylamide (KPAM)	Shale-inhibiting polymer, viscosity enhancer	Improves wellbore stability; effective in low-solids muds	Overuse increases yield stress and pumping energy
Acrylamide-co-AMPS Copolymer	HPHT filtrate-control additive	Good thermal (≥200 °C) and salt resistance; stable in brine	Relatively expensive; requires dispersion control
Polysaccharide–Nanocomposite Blends (e.g., XG–SiO_2_)	Combined rheology and filtrate control	Enhanced thermal stability (>200 °C) and improved shale inhibition	Preparation complexity; dispersion stability must be maintained

**Table 2:** A comparison of polymer–nanoparticle integration methods, detailing their compatibility, processing characteristics, and resultant fluid performance.

Method	Polymer Type	Nanoparticle Compatibility	Dispersion Quality	Scalability	Impact on Stability & Shale Inhibition
Solution Mixing	Water-soluble polymers (PAM, PAC, CMC)	SiO_2_, TiO_2_, GO	Good at lab scale; solvent removal may cause agglomeration	Moderate	Ensures high uniformity; improved filtrate control, but limited long-term NP stability
Melt Mixing	Thermoplastics and modified gums	SiO_2_, ZnO, CNTs	Good physical dispersion; thermal shear may cause NP clustering	High	Produces durable, temperature-resistant composites; effective for rheology but moderate shale inhibition
In situ Polymerization	Acrylamide, AMPS, or acrylic copolymers	TiO_2_, ZnO, GO, α-LAP	Higher chemical bonding and NP immobilization	Moderate to high	Best dispersion and stability; strong barrier formation and shale inhibition under HPHT

**Table 3:** Overview of current studies demonstrating improvements in the rheology of drilling fluids via PNCs.

Polymer Used	Nanofiller Used	Tested Rheology Parameters	Test Conditions	Main Findings	Benefits	Reference
ANDP (AM, AMPS, DMDAAC)	SiO_2_, Metal Oxides	PV, AV, YP, GS	150–200 °C, 500 psi	At 200 °C, ANDP improved AV, PV, and YP over the base fluid.	High thermal stability and salt resistance prevent clay aggregation.	[54]
C-g-AA-NH2	KCl	PV, AV, YP	25 °C, 100 psi	2 wt% AA-C-g-AA-NH2 outperformed 10 wt% KCl in rheology.	Effective KCl replacement at a lower dosage.	[55]
SP-GO	GO	PV, AV, YP, GS	78–250 °F	0.4 wt% gave best performance; improved PV and YP.	Lubricates and enhances rheology in salty, high-temp conditions.	[56]
PEG-SiO_2_ NC + SDS	SiO_2_, SDS	PV, AV, YP, GS	78–250 °F, 500 psi	At 1.5 g, boosted PV by 75%, YP by 100%, and AV by 81.8%.	SDS enhances dispersion and thermal stability.	[57]
PEG–SiO_2_	SiO_2_	PV, AV, YP, GS	78–250 °F, 500 psi	Slight decrease in AV/PV vs. base fluid, but good thermal performance.	Stable rheology at high temperature and pressure; compatible with mud systems.	[58]
PAAG NC	SiO_2_	PV, AV, YP	25–180 °C, 500 psi	1.0 wt% improved rheology by 46–80%.	Good salt resistance and thermal stability up to 240 °C.	[59]
Hydrophobic polymer NC	Bentonite	PV, YP, GS	25 °C, 100 psi	PNCs improved viscosity and structure due to the interaction with bentonite.	Promotes uniform dispersion and consistent fluid behavior.	[60]
PP-SiO_2_ vs. PHPA	SiO_2_	AV, PV, YP, GS	25–150 °C, 500 psi	PNCs exhibited greater YP and thermal performance compared to PHPA.	Sustains properties under heat without degradation.	[61]
PVP/SWCNTS	CNTs	AV, PV, YP, GS	25 °C, 100 psi	Small drops in AV and PV; shear-thinning behavior.	Good mud stability with little performance change.	[62]
CuO/PAM NC	ZnO	AV, PV, YP, GS	25 °C, 100 psi	Big rheology improvements in fresh and salt water.	Stable and effective across fluid types.	[63]
XG–SiO_2_	SiO_2_	AV, PV, YP, GS	25–120 °C, 500 psi	Outperformed XG and other base muds.	Strong rheological performance even at HPHT.	[64]
PP-SiO_2_–NH2	SiO_2_	AV, PV, YP, GS	25 °C, 100 psi	Improved PV from 11 to 15 mPa·s and YP from 9 to 14 Pa.	Good thermal resistance and rheology stability.	[65]
PSt MMA nano clay	Clay (Laponite)	AV, PV, YP, GS	25 °C, 100 psi	AV/PV increased by 44–51%.	Stable up to 120 °C; enhances mud rheology.	[66]
Nano-LS-g-PAM-AMPS	SiO_2_	AV, PV, YP, GS	200 °C, 600 psi	Strong AV/PV/YP increase with thermal/salt resistance.	Durable at high temperatures and salinity.	[67]
Laponite NC polymer	Laponite	AV, PV, YP, GS	Up to 260 °C	Maintains viscosity after 72 h of aging.	Extreme thermal stability.	[68]
Laponite copolymer	Laponite	AV, PV, YP	210 °C, 500 psi	Stable AV (19 cP), PV (15 cP), YP (8 lbf).	High-temp stability.	[69]
PEG–NS	SiO_2_	PV, YP, GS	78 °F	PV improved by 14% and YP by 50%.	Stable up to 200 °C.	[70]
SMA/SiO_2_	SiO_2_	PV, YP, GS	78 °F	Minor rheology change; filtration control improved.	Reasonable fluid loss control.	[71]
CNT/ZnO/polymers	CNTs, ZnO	AV, PV, YP, GS	Up to 400 °F	Rheology remained stable post-aging.	High-temp durability.	[72]
Core–shell NC	SiO_2_	PV, AV, GS	120 °C, 100 psi	Rheology close to commercial CMC.	Reasonable control and competitiveness.	[73]
MES/Polystyrene NC	Polystyrene	PV, AV, GS	150 °C, 500 psi	Exhibits shear thinning and uniform velocity.	Stable thermal and flow behavior.	[74]
TiN NPs in KCl	Titanium Nitride (TiN)	Cof, AV, PV, YP, GS	25 °C	YP improved by 122%, PV by 17%.	Good cutting, transport, and hole cleaning.	[75]
Hydrophobic PEG–SiO_2_	SiO_2_	PV, YP, GS	250 °F	AV/YP/PV all increased.	Improved NPs integration and fluid loss control.	[76]
PAM–PEG–SiO_2_	SiO_2_	PV, YP, GS	95 °C, 500 psi	AV and YP improved greatly.	Enhances stability up to 95 °C.	[77]
PAM–ZnO NC	ZnO	AV, PV, YP, GS	80–150 °F	18–17% increases in PV and YP.	Works well even at low dosages.	[78]
EDA–G graphene NC	Gr	PV, AV, YP, GS	150 °C, 500 psi	Good rheology; salt- and calcium-resistant.	Effective in extreme salinity.	[79]
SFDL core–shell NC	SiO_2_	PV, AV, YP, GS, Cof	Up to 446 °F	Massive gains in AV, PV, and YP; Cof dropped significantly.	Prevents polymer breakdown at very high temperatures.	[80]
TiO_2_–PAM NC	Titanium Dioxide TiO_2_	PV, AV, YP, GS	25 °C	273% PV increase with 14 g PNCs.	Good cross-linking and high stability.	[81]
PAM/Al_2_O_3_ NC	Alumina (Al_2_O_3_)	PV, YP, AV, GS	150 °C, 500 psi	PV exceeded 300 cp at a 4 wt% concentration.	Long-term mud stability and compatibility.	[82]
ZnO–Clay NC	ZnO, Clay	PV, YP, AV, GS	110–370 °F, up to 18,500 psi	PV and YP were greatly enhanced at 370 °F.	Good under extreme HPHT.	[83]

**Table 4:** Current analysis of PNCs materials utilized as shale rheological properties, and viscosities in (WBMs) drilling.

Material Used (PNCs)	Polymer Used	Nanofillers Used	Key Findings	Shale Inhibition Mechanism	Reference
PNSC + NH_2_	PNSC	Nanohydroxide (NH_2_)	The percentage of shale retained after the hydration test was 75.3% with PNSC + NH_2_, compared to 33.6% with base mud and 58.4% with KCl.	Uses physical plugging and hydrogen bonding to reduce water contact between the shale and the fluid.	[87]
PEG-NS	Polyethylene glycol (PEG)	SiO_2_	PEG-NS exhibited larger plugging capacity and retarded shale hydration, with slower pressure growth than JHC.	Physical plugging and disruption of hydrogen bonds to reduce shale hydration and water absorption.	[88]
AR/SiO_2_ NC	Acrylic Resin (AR)	SiO_2_	Reduced API FL by 53.6% and API FCT by 23.1%, proving effective in inhibiting shale hydration.	Forms substantial physical barriers by sealing microcracks and nanopores in shale through AR/SiO_2_’s core–shell structure.	[89]

**Table 5:** Recent studies demonstrate that adding PNCs to WBMs reduces fluid loss.

Materials Used	Test Conditions	Main Results	Reference
Used a polymer mix (AM, AMPS, DMDAAC) with nano-laponite	Tested in both freshwater and saltwater mud at 150–200 °C	2% PNCs reduced fluid loss in saltwater mud from 280 mL to 72 mL after heating for 16 h.	[92]
Combined salt-based polymer with GO	Concentrations of 0.1–0.5% at 78 and 250 °F	The blend improved fluid filtration performance at both temperatures.	[93]
Mixed polypropylene beads with silica	Used 8 g PP and 1 g silica at 78 and 250 °F	Reduced fluid loss by 24% and slightly decreased filter cake thickness.	[94]
Created a nanocomposite using AM, AMPS, NVP, and GO	Evaluated at 25, 150, and 180 °C	1% dosage maintained strong filtration control even with high salt content (up to 25%).	[23]
Blended PVP with CNTs	Tested 1% and 5% at 25 °C	Achieved 18–23% reduction in fluid loss.	[95]
Used polypropylene and PHPA with modified silica	Tested at 25 and 120 °C	Improved high-temperature stability and reduced fluid loss by about 18%.	[51]
Combined PAM-AMPS with nano-lignite	LPLT: 78 °F/HPHT: 356 °F	Aging produced good fluid loss results (7.1 mL LPLT, 30 mL HPHT)	[96]
Used PSt MMA and nano-clay	Tested at 78 and 250 °F	Reduced fluid loss by up to 65% under high-temperature conditions.	[97]
Developed a copolymer with laponite	HPHT at 356 °F and 500 psi	Halved the fluid loss from 34 mL to 16 mL.	[8]
Combined PAM with copper oxide	Used 1–10 g doses at 25 and 120 °C	Lowered fluid loss and produced a thinner filter cake.	[98]
Created a nanocomposite with PEG and nano-silica	1% tested at 78°f	Cut fluid loss by over 15%.	[34]
Mixed methyl ester sulfonate with nano-polystyrene	Tested at both low (78 °F) and high (250 °F) temperatures	Reduced fluid loss by 22% at LPLT and 61% at HPHT due to a synergistic effect.	[34]
Blended SMA with silica	2% tested at 78 °F	Reduced API fluid loss by 22%.	[99]
Combined CNT, ZnO, and synthetic polymers	Tested up to 400 °F	Maintained stable mud and created thin, less permeable filter cakes.	[100]
Used carboxymethyl cellulose with polystyrene	LPLT test at 25 °C	It resulted in the lowest fluid loss volume in this category.	[101]
Combined PEG and silica	Tested at 78 and 250 °F	2% dosage performed well even after thermal aging.	[102]
Used PAM with ZnO	Tested at 78 °F and 150 °F	Reduced fluid loss by 12.7% (LPLT) and 23% (HPHT).	[21]
Created PAM-silica nanocomposite	0.1–1.0% tested at up to 203 °F	0.7% dosage provided stable and effective filtration.	[103]
Used AM, AMPS, MA, and St with silica	Tested from 78 °F to 446 °F	Withstood extreme conditions and effectively controlled fluid loss.	[104]
Blended PAM with titanium dioxide	Doses from 1–14 g at 25 °C	Reduced fluid loss from 53 mL to 19 mL at the highest dose.	[105]

**Table 6:** Current analysis of PNCs materials utilized as fluid loss agents and viscosities in WBM drilling.

Material Used (PNCs)	Polymer Used	Nanofillers Used	Key Findings	Shale Inhibition Mechanism	Reference
PAM-SiO_2_ NC	PAM	SiO_2_	Achieved the highest shale stability (86.6%) over 48 h, outperforming commercial inhibitors (74.7%) and KCl (49.2%).	Disrupts hydrogen bonds in water through PAM-SiO_2_ NC’s OH groups and physically blocks microcracks and nanopores in shale.	[109]
C-g-AA-NH_2_	Acrylic acid (AA)	Aminomethyl, Amyl	Achieved a high anti-swelling ratio of 95.2% with AA-AAm-C-Amyl, surpassing KCl (55.4%).	Combines carbon core nanopore plugging and strong polymer adsorption to inhibit swelling.	[110]
AP-ZnO NC	Acrylic Polymer (AP)	ZnO	Shale recovery improved to 97% with AP-ZnO NC compared to 81% with BF; the swelling rate reduced from 14.29% to 6.69%.	ZnO reduces mud invasion by plugging pores and cracks, while AP encapsulates the shale surface, preventing water interaction.	[111]

## Data Availability

No new data were created or analyzed in this study. Data sharing is not applicable to this article.

## Abbreviations

AV	Apparent Viscosity
CMC	Carboxymethyl Cellulose
CNT	Carbon Nanotube
FCT	Filter Cake Thickness
GG	Guar Gum
GS	Gel Strength
HPAM	Hydrolyzed Polyacrylamide
HPHT	High-Pressure High-Temperature
HEC	Hydroxyethyl Cellulose
LCM	Lost Circulation Material
NP(s)	Nanoparticle(s)
OBM	Oil-Based Mud
PAC	Polyanionic Cellulose
PAM	Polyacrylamide
PHPA	Partially Hydrolyzed Polyacrylamide
PNC(s)	Polymer Nanocomposite(s)
PV	Plastic Viscosity
ROP	Rate of Penetration
SiO_2_	Silica
WBM	Water-Based Mud
XG	Xanthan Gum
WBMs	Water-Based Muds
OBMs	Oil-Based Muds
PNCs	Polymer Nanocomposites
HPHT	High-Pressure & High-Temperature
KPAM	Potassium Polyacrylamide
YP	Yield Point
CoF	Coefficient of Friction
GO	Graphene Oxide
CNTs	Carbon Nanotubes
Gr	Graphene
QDs	Quantum Dots
MSNs	Mesoporous Silica Nanoparticles
FESEM	Field-Emission Scanning Electron Microscopy
FTIR	Fourier-Transform Infrared Spectroscopy
